# IL-17: an important pathogenic factor in endometriosis

**DOI:** 10.7150/ijms.71972

**Published:** 2022-04-11

**Authors:** Jia-Lu Shi, Zi-Meng Zheng, Min Chen, Hui-Hui Shen, Ming-Qing Li, Jun Shao

**Affiliations:** 1 Laboratory for Reproductive Immunology, Hospital of Obstetrics and Gynecology, Fudan University, Shanghai 200080, People's Republic of China.; 2 NHC Key Lab of Reproduction Regulation (Shanghai Institute for Biomedical and Pharmaceutical Technologies), Hospital of Obstetrics and Gynecology, Fudan University, Shanghai 200080, People's Republic of China.; 3 Shanghai Key Laboratory of Female Reproductive Endocrine Related Diseases, Hospital of Obstetrics and Gynecology, Fudan University, Shanghai 200080, People's Republic of China.

**Keywords:** IL-17, endometriosis, cytokine, inflammatory, Th17 cell

## Abstract

Interleukin-17 (IL-17) is known as a Th17-cell-derived proinflammatory cytokine, which plays a pivotal role in several inflammatory and autoimmune diseases such as systemic lupus erythematosus (SLE), rheumatoid arthritis, and psoriasis. Emerging evidence has shown that IL-17 is linked to endometriosis, although the etiology of endometriosis is still unknown. The IL-17 expression is up-regulated in serum, peritoneal fluid (PF) and endometriotic lesions from patients with endometriosis but the related regulation mechanisms are complex and obscure. Meanwhile, the specific roles of IL-17 in endometriosis are also worthy of further exploration. Through the integration and summary of literature, we conclude that the secretion of IL-17 increases under the regulation of ectopic microenvironment and other factors, and then IL-17 is deeply involved in endometriosis in the regulation of immune microenvironment, the invasion and growth of ectopic lesions, and so on, which implies its therapeutic value in this disorder.

## Introduction

The IL-17 is secreted by the CD4^+^ T helper 17 (Th17) cells and regarded as the signature cytokine of a distinct cluster of these cells, which was discovered in 1999 using T-cell clones from the joints of patients with rheumatoid arthritis 1-4. The development of Th17 cells is distinct from the development of Th1, Th2 and regulatory T cells and requires specific transcription factors and cytokine requirements, such as transforming growth factor-β (TGF-β), combined with IL-6 or IL-21 and the transcription factor, retineic-acid-receptor-related orphan receptor gamma (RORγt). As for their particular expression of the “master” transcription factor RORγt, it is then activated by the IL-12 family cytokine IL-23, and the resulting “IL-23-IL-17 axis” was found to function as a critical driver of autoimmune disease 5, 6, 4, 7-16. To date, the IL-17 family of cytokines contains 6 structurally related cytokines (IL-17A, IL-17B, IL-17C, IL-17D, IL-17E and IL-17F) that share sequence homology, and their 5 corresponding receptors (IL-17RA, IL-17RB, IL-17RC, IL-17RD and IL-17RE) present on the surface of cells. These IL-17 receptors subunits adopt a shared cytoplasmic motif termed a ''SEFIR'' (SEF/ IL-17 receptor), which is analogous to the toll-IL-1 receptor (TIR) domain expressed in toll-like receptor (TLR) and IL-1 receptor family members 10, 17-19. IL-17A is the first described member of this family and also the best characterized one. It was once believed to be primarily produced by Th17 cells 20, 21, 19. In fact, IL-17A and IL-17F exhibit high sequence similarity and can form homodimers and heterodimers to signal, and they also signal through the same receptor complex, so they largely share biological functions, with IL-17A being more potent than IL-17F. While the other four IL-17 isoforms only exist as homodimers 3, 22, 18, 23.

Since IL-17 is reported to be conserved in evolution 24-28, its host-protective attributes can play diverse roles both in immune-protection and also immunopathology. As for immune-protective functions, IL-17 exerts its function as a key mediator of mucosal surveillance and barrier integrity through maintaining epithelial integrity, promoting the production of antimicrobial factors and regulating the recruitment and generation of neutrophils 29-35, 22, 36, 37. However, IL-17 also has been increasingly implicated as a driver of immunopathology in settings of autoimmunity, cancer and chronic inflammation 38-41. Take IL-17A as an example, it has been recognized in its critical role in the promotion of disease progression, pathogenesis of autoimmune diseases, tumors, mechanical injury, infection, obesity and chronic inflammatory disorders 20, 10, 42, 18, 43, 19.

Endometriosis is a common estrogen-dependent inflammatory gynecological disease and is defined as the presence of functional endometrial glands and stroma outside the uterine cavity.^44^-^46^ This disorder is similar to malignancies in some ways: progressive and invasive growth, a tendency to metastasize and recurrence. Although the physiopathology of endometriosis is not completely understood and several theories have been proposed to explain it, it is well established that this pathogenesis is closely related to the immune system. The defective immune responses may include increased levels of activated peritoneal macrophages and various proinflammatory cytokines, abnormal T- and B-lymphocyte activation, reduced natural killer cell activity, and the production of various autoantibodies 47, 48, 44, 49-56. All these activities not only fail to effectively clear discarded endometrial tissues but may actually allow development of chronic inflammation and even a hyperinflammatory state, which can help endometrial cells to escape immunosurveillance and also use inflammatory mechanisms to promote their growth within the peritoneal cavity. The amount of misplaced endometrial tissues will in turn overwhelm the resident and recruited immune cells, leading to dysfunctional in their ability 57, 50. And as is reported in many previous studies, the IL-17 family plays a pivotal role in the pathogenesis of endometriosis 58, 49, 59-61.

In this review, we attempt to outline the roles of IL-17 in endometriosis, present the regulatory mechanism of IL-17 expression in endometriosis, identify the biological function (regulation of ectopic endometrial lesions, recruitment and function regulation of immune cells, and angiogenesis) of IL-17 in endometriosis, and discuss prospects in the potential treatment of these patients as well.

## Expression of IL-17 and its receptors

With the deepening of the research on cytokines and endometriosis, elevated levels of IL-17 in endometriosis have been reported and confirmed more widely, especially in the early stages of the disease 62-64, 42, 60, 61. Further investigations have documented that IL-17 is produced not only by Th17/ThIL-17 cells, but also by activated CD8^+^ T cells, γδ T cells, NK cells, neutrophils as well as mast cells 65-72. For the first time, Zhang and his colleagues demonstrated higher IL-17 levels in the PF of patients with endometriosis. Meanwhile, there was a correlation between the concentration of IL-17 in PF and progression of the disease, and the concentrations of IL-17 in PF were significantly higher in the patients with minimal/mild endometriosis than those with moderate/severe endometriosis and those without endometriosis. This study also suggested the concentration of IL-17 in PF was associated with endometriosis-related infertility 61. In line with Zhang's study, increased amounts of IL-17 was later demonstrated in the PF of women with endometriosis by some other teams 62, 63, 73, 74, 42, 60. Bungum et al. also found high expression of IL-17E in PF, but unlike Zhang's team, they did not find an association between IL-25 (also called IL-17E) levels and the stage of endometriosis 62. They speculated that this result could be due to the fact that the inflammatory response seems to be low at more severe stages of endometriosis, just as Salmeri et al. suggested 75. Besides, some other reports even demonstrated that in the context of endometriosis, IL-17A was elevated in the plasma 76 and PF of women with endometriosis compared to controls and that endometriotic lesions produce IL-17A 42, 60. Sabbaghi et al. declared that a similar elevation in IL-17A level was observed both in blood serum and follicular fluid (FF) when endometriosis and infertility co-exist 77. In Ahn's research, though they did not find a significant difference in the PF concentration of IL-17A between women with endometriosis and without disease, they found it in the plasma samples. Additionally, in their study, immunohistochemistry revealed the localization of IL-17A-positive cells in the stroma and surrounding the vasculature in matched eutopic endometrium and ectopic lesion samples from women with endometriosis. Thereout, they suspected it was possible that IL-17A was primarily generated by tissue-resident immune cells and as such may not be detectable in the PF 78.

In addition, Hirata et al. successively reported that endometriotic stromal cells (ESCs) expressed IL-17RA 64 and IL-17RC 79. In 2008, this group first examined presence of IL-17A-positive cells in endometriotic tissues and Th17 cells in peritoneal fluid mononuclear cells (PFMCs) 64. Then they also demonstrated expression of IL-17F in mononuclear cells from endometriotic lesions (EMMCs) 79.

A recent study, performed by Gogacz et al., described the increased percentage of Th17 cells in the PF in comparison with peripheral blood (PB) in endometriotic patients. And their data also showed that the percentage of Th17 cells in PF corresponded with the severity of endometriosis. In severe endometriosis, the percentage of Th17 cells in PF was higher than with early (I/II stage) endometriosis 80. This correlation was also demonstrated by other researchers 81. Liu et al. further showed that the percentage of IL-17 and Th17 cells were both increased in peritoneal fluid mononuclear cells (PFMCs) of patients with endometriosis 82. It is also newly demonstrated the abundance of CD8^+^ T cells and CD56^+^ NK cells with enriched IL-17 signalling pathway in the eutopic endometria of women with endometriosis 83.

However, there are several papers reporting that they did not find any relationship between the level of IL-17 and endometriosis 84, 85. But some of them still found IL-17/IL-10 and IL-17/IL-23 ratios were respectively increased in PF and serum samples from endometriosis group. And the increased IL-17/IL-23 ratio was also found in the periphery of endometriosis women, which was explained because IL-17 was antagonized by anti-inflammatory cytokines such as TGF-β1 in the latter stages of the disease (**Table 1**) 85.

By considering the above-mentioned facts, it is prevalent that the level of IL-17 rises in endometriosis patients, commonly detected in PF and blood. Due to the effects of anti-inflammatory factors and other unknown factors, this concentration is not necessarily proportional to the progression of the disease. It is possible that continuous dynamic detection of concentration changes will provide more valuable hints, which require further large sample studies. Besides, in view of the differences in sample size, measurement methods and sampling among different studies (sampling at different times of the menstrual cycle, measure differences in samples such as tissues or cells and etc.), there must be inevitable differences in different research results.

## Regulatory mechanisms of IL-17 expression in endometriosis

### Estrogen

Deena Khan's team found that estrogen not only enhanced the levels of IL-17 and intracellular IL-17^+^ cells but also upregulated the IL-17-specific transcription factor, ROR**γ**t, in activated splenocytes in wide type mice. They also suggested estrogen upregulates IL-17 induction in autoimmune mice 86. Similarly, Newcomb et al. provided evidence that 17β-estradiol (E2) and progesterone (P4) increased IL-17A production from Th17 cells, by decreasing let-7f miRNA expression and increasing IL-23R expression 87. While other* in vitro* studies reported the production of IL-17 was under the negative regulation of E2. These variable findings might be due to different T-cell activation and differentiation protocols 88-93. Taken together, these findings confirmed that estrogen is involved in the expression of IL-17, although there is no direct evidence that the site of action is endometriosis. Furthermore, Ning's findings showed IL-17A participated in CD68^+^CD163^+^ macrophage-stimulated endometrial cancer cell proliferation by regulating the estrogen receptor alpha (Erα) pathway 94. According to the above analysis, we suppose that estrogen may well be involved in the expression of IL-17 in the microenvironment of endometriosis and prefer it to be a positive moderator, which still warrants further investigation.

### Cytokine

It is extensively acknowledged that some cytokines also participate in the production of IL-17 in endometriosis. Bungum et al. proposed that the pathophysiology could involve macrophages producing IL-1β and tumor necrosis factor-α (TNF-α), found in PF from women with endometriosis 95, which stimulates production of Regulated on Activation, Normal T cell Expressed and Secreted (RANTES) and Monocyte Chemotactic Protein-1 (MCP-1) 96, 97. RANTES and MCP-1 may be responsible for recruiting macrophages, producing Histamine Releasing Factor (HRF), into endometriotic implants. In turn HRF might induce production and release of IL-4 and maybe IL-25 from mast cells 62. Current evidence largely suggests that IL-23 is responsible for the differentiation and expansion of Th17/ThIL-17 cells, and so it is viewed as an important IL-17 inducer to regulate the expression of IL-17 98, 99, 35, 100. Additionally, Chang's group concluded that under the stimulation of IL-6 and TGF-β, signal transducer and activator of transcription 3 (STAT3) may be activated in naive T cells, which further promotes RAR-related orphan receptor C (RORC) and IL-17A transcription, and induces IL-17A production. And IL-27 was considered to induce the IL-10 and IL-17A double-producing Th17 cells in endometriosis. In advanced endometriosis, the formation of a c-Maf, RORγt and Blimp-1 complex triggered by IL-27 contributes to the expansion of IL-10-producing Th17 cells. Hence IL-27 is regarded as a pivotal regulator in endometriotic immune tolerance by triggering Th17 cells to produce IL-10 and IL-17A and promoting the rapid growth and implantation of ectopic lesions 101. TGF-β1, a kind of anti-inflammatory cytokines with a myriad of functions including cell differentiation, proliferation, migration, angiogenesis and vasorelaxation 102, 103, is found to support both Th17 and Treg cells differentiation in a dose dependent manner. And in higher concentrations, the immune response will shift toward Treg cells 104. Therefore, Tarokh et al. assumed that higher concentrations of TGF-β1 might regulate the inflammation in the patients via reducing IL-17 concentration in the later stage of endometriosis 85. In a recent study, the researchers found that treatment with anti-bone morphogenetic protein 1 (anti-BMP1) antibodies dose-dependently increased lesion volume in mice with endometriosis, reduced IL-17 and IL-1β levels 105. This may suggest that BMP1 is involved in the regulation of IL-17 expression although further study is needed to confirm it. Given all this, the role of cytokines, in the regulation of IL-17 expression in endometriosis is beyond doubt. However, further studies are still needed to determine whether the specific regulatory mechanisms are intersected or the effects of various cytokines are independent. Inhibition of positive factors and promotion of negative factors may help to reduce the concentration of IL-17, thus providing new ideas for the treatment of endometriosis.

### LnRNA and microRNA

Gene-level studies on the regulation of IL-17 expression in endometriosis are also under way. Zhi et al. proved that lack of immediate early response gene (IER3, also called IEX-1), belonging to the group of genes rapidly activated during inflammation, promoted Th17 differentiation and then increased IL-17A production 106. And IER3, predicted by bioinformatics software, is one of the target genes of miR-342-3p which has been found to be highly expressed in serum of woman with EMS 107. Besides, LncRNA H19 was also successively detected in women with EMS and found lower expression in the eutopic endometrium of women with EMS with its mechanism in reducing the proliferation of ESCs 108, 109. Recently, Liu et al. first confirmed miR-342-3p could negatively regulate IER3 expression. And they also demonstrated that LncRNA H19 over-expression could decrease IL-17 secretion, suppress Th17 differentiation and ESCs proliferation through inhibiting miR-342-3p 82. It is plausible that given the limited exposure reported to date, miR-342-3p has a positive promoting effect on IL-17, while LncRNA H19 plays a detrimental role.

### Hypoxia

Retrograde menstruation has been proposed and well accepted to be a crucial constituent for the development of the etiology of endometriosis. According to this theory, shed-off endometrial tissues first lost blood supply and may well face hypoxic stress. Consequently, the ectopic lesion in endometriosis is indeed an anoxic environment. Hypoxia-inducible transcription factor-1 (HIF-1), the oxygen-sensitive transcription factor, is key transcriptional regulator of hypoxia-associated genes to adapt to decreased availability of O_2_ 110. Dang et al. once observed a higher proportion of IL-17A^+^ cells in hypoxic compared to normoxic culture conditions. And their results demonstrated that HIF-1 plays a dual role in regulating IL-17A transcriptional activity by directly activating RORγt transcription and then collaborating with RORγt at the IL-17A promoter to recruit p300, thus generating a permissive chromatin structure. Meanwhile, Th17 differentiation is enhanced under hypoxia in a HIF-1α-dependent manner. Therefore, HIF-1α activity is suggested to represent a major mechanism by which the hypoxic conditions associated with inflammation can promote Th17 differentiation 111. It is also proved that hypoxia and HIF-1α overexpression in in rheumatoid arthritis potentiated RASF-mediated expansion of inflammatory Th1 and Th17 cells, leading to proinflammatory interferon-γ (IFN-γ) and IL-17 production 112. In addition, it has been found that the abundance of HIF isoforms was mechanistically linked to elevated IL-1β and IL-17 in sarcoidosis 113. Taken together, IL-17 is regulated by HIF-1 and hypoxia. However, it is a pity that there may be scarce direct evidence that hypoxia or HIF-1 regulates IL-17 expression in endometriosis up to date. Further research is needed on whether the same regulatory mechanism exists in endometriosis.

## The biological functions of IL-17 in endometriosis

### Regulation of ectopic endometrial lesions

Based on an analysis of the literature, we suspect that the regulation of IL-17 on ectopic endometrial is mainly likely to trigger its invasion, implantation, growth and proliferation during the early stage of disease, that is IL-17 might be more important in the initiation, but not in the later process of endometriosis 64, 114, 85. Ahn et al. reported that IL-17A mainly promoted proliferation and invasion, and restricted the adhesion of ESCs, thereby accelerating the growth, implantation and dissemination of an ectopic lesion *in vitro* and *in vivo* 78, which is also verified by Chang's team 101. IL-17A stimulated propagation of ESCs may be partially attributable to its mitogenic effect and the increased production of IL-8 induced by IL-17A 64, since IL-8 has been shown to facilitate the proliferation of endometrial stromal cells 115. And another member of the IL-17 family, IL-17F, had a similar function in stimulating the secretion of IL-8 and the expression of COX2 in ESCs and it is speculated IL-17F may promote endometriosis through these mechanisms 79.

In addition, IL-17 has also been found to enhance both the production and secretion of IL-1β by peritoneal macrophages 116. It has been reported that IL-1β induces the production of IL-8 and vascular endothelial growth factor (VEGF) 117. Therefore, elevated concentrations of IL-17 in patients with endometriosis may imply an increased hypervascularisation, leading to possible facilitation of the implantation, proliferation and establishment of early endometriotic lesions 61.

Furthermore, according to Khan's finding, endometriosis is relevant to Treg- and Th17-cell alteration causing survival and implantation of ectopic endometrial lesions in the initial stage of the disorder, with consequent progression toward the advanced stage 73.

### Recruitment and function regulation of immune cells

Hirata et al. have shown that one of the pathogeneses of endometriosis by which IL-17A is involved in is the secretion of IL-8 64, 114, 79. While pleiotropic functions of IL-8, such as chemoattraction and activation of neutrophils, are clear and suggested to promote endometriosis 118-120. Additionally, IL-17 can enhance granulopoiesis by stimulation of granulocyte colony-stimulating factor (G-CSF) and granulocyte macrophage colony-stimulating factor (GM-CSF) 121. Takamura et al. suggested that IL-17A produced by neutrophils stimulates growth-related oncogene-α (Gro-α) secretion from EoSCs, thereby recruiting more neutrophils and inducing perpetuating inflammation in endometriosis 122. Gro-α is known as a powerful activator of neutrophils in its ability to induce chemotaxis, a rise in intracellular free calcium, exocytosis, and the respiratory burst in neutrophils 123, 124, 120. While neutrophils are suggested to secrete VEGF under inflammatory milieu, trigger angiogenesis in the early stage of endometriosis, generate reactive oxygen species (ROS) at endometriotic sites and impose oxidative stress that affects the development of endometriosis 125, 126.

Furthermore, macrophages are also thought to be a pivotal player in promoting endometriosis 54. It is reported that IL-17A is chemotactic for macrophages via its receptor, IL-17RA, and can also induce M2 polarization in lung cancer 127, which is also demonstrated in endometriosis. These researchers propose IL-17A is involved in macrophage recruitment and may be indirectly polarizing SPM into a pathogenic M2 phenotype by first interacting with the endometriotic lesion 42. Indeed, M2 macrophages have been proved to mediate processes such as extracellular matrix (ECM) reconstruction and vascularization, which are associated with the progression of endometriosis 128-130. And increased production of TNF caused by the activated macrophages and biological effect of IL-17 on endometrial cells accelerates the occurrence of endometriotic lesions accompanied by infertility and unexplained pelvic pain. Besides, IL-17 has been shown to induce the production of nitric oxide synthase 2 (NOS2) and nitric oxide (NO) by peritoneal macrophages 131. Recent work suggested that increased levels of IL-17 in PF from the endometriosis patients with infertility may induce the production of NOS2 and NO by peritoneal macrophages, which adversely affect female reproductive system, sperm, embryos, implantation and oviductal function. These abnormalities likely all impact fertility, leading to the infertility or subfertility observed in the patients with endometriosis 61, just like the toxic effects of those increased concentrations of Th1 cytokines such as TNF-α, IL-1β, IL-4, IL-6, IL-12, and IFN-γ in women with endometriosis 132-139. It is also showed that IL-17 should be inhibited by the emergence of decidual stromal cells and placentation, which enables the evolution of embryo implantation and ongoing pregnancy 140. Herein, it can be inferred that the elevated IL-17 level is related to poor reproductive outcome not only in chronic endometritis 141 but also in endometriosis.

Overall, IL-17 could be the stimuli that mediates the recruitment and activation of immune cells such as macrophages and neutrophils to facilitate the immune escape of ectopic endometrial cells, promote the progress of endometriosis, and contribute to the unexplained infertility.

### Angiogenesis and vasculogenesis

The survival of endometriotic implants on the peritoneal membrane within the peritoneal cavity relies on the establishment of blood supply for the provision of oxygen and nutrients to the developing lesions. And endometriotic lesions are densely vascularized. All these fuel the notion that mechanisms of angiogenesis and/or vasculogenesis may be utilized by endometriosis to establish its own vascular network to sustain its survival 142. Angiogenesis refers to a complex process of new blood vessel formation from previously existing vessels with endothelial cell proliferation 143, 144. While vasculogenesis refers to a process of de novo formation of blood vessels arising from migration, proliferation, and incorporation of angioblasts or endothelial progenitor cells (EPCs) from the bone marrow 145.

**Figure 1:**
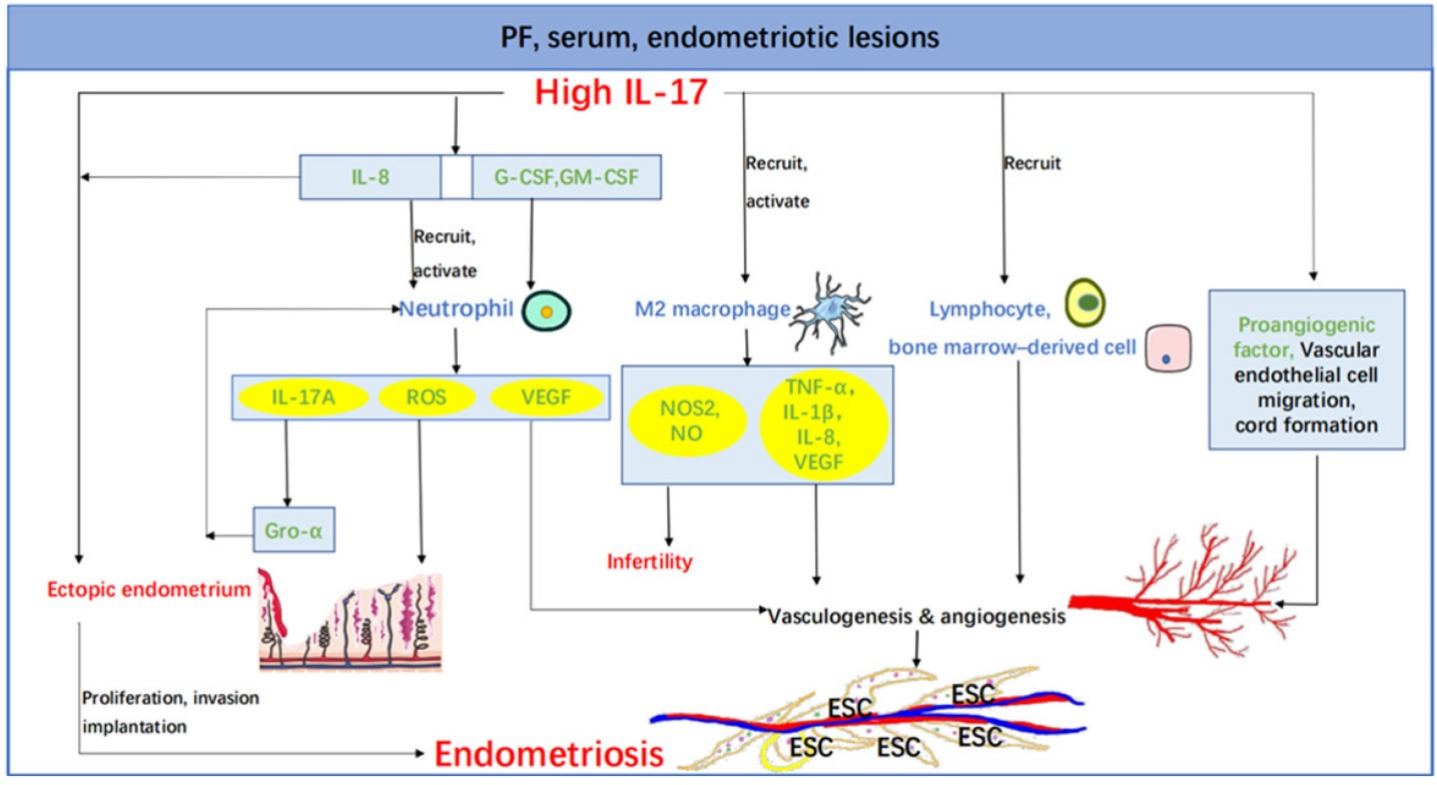
** The role of IL-17 in endometriosis.** Increased IL-17 in PF, serum, endometriotic lesions and etc. leads to the proliferation, invasion and implantation of ectopic endometrium partly by recruiting and activating neutrophil and M2 macrophage. And the macrophage secretes NOS2 and NO, giving rise to co-existence of endometriosis and infertility. IL-17 also acts directly on its own and indirectly through IL-8. The promotion of vasculogenesis and angiogenesis is another important role of IL-17, which can act through a variety of pathways, such as stimulation of vascular endothelial cell migration and cord formation, recruitment of lymphocytes and bone marrow-derived cells and inducing elaboration of a variety of proangiogenic factors (e.g., VEGF, IL-1β, TNF-α, and IL-8). Abbreviations: PF, peritoneal fluid; IL, interleukin; G-CSF, colony-stimulating factor; GM-CSF, granulocyte macrophage colony-stimulating factor; ROS, reactive oxygen species; VEGF, vascular endothelial growth factor; NOS2, nitric oxide synthase 2; NO, nitric oxide; Gro-α, growth-related oncogene-α; TNF-α, tumor necrosis factor-α; ESC, endometriotic stromal cell.

Numasaki et al. reported that IL-17 promotes angiogenesis via inducing elaboration of a variety of proangiogenic factors that lead to the imbalance between angiogenesis activators and inhibitors present within the vascular microenvironment and triggers vasculogenesis via stimulation of vascular endothelial cell migration and cord formation. IL-17, on the other hand, stimulates production of proangiogenic factors in fibroblasts and promotes fibroblast-induced neovessel formation in inflammation 146. Another study suggests that IL-17A has the potential to enhance vascularization of the lesion through VEGF-and IL-8-mediated pathways. Their data also prove a potential involvement of IL-17A in mediating neoangiogenesis and recruitment of lymphocytes and bone marrow-derived cells to the site of lesion development 78. Moreover, as mentioned above, IL-17 has the capacity to activate macrophages and neutrophils. Then the secretory products such as TNF-α, IL-8, and VEGF secreted by activated macrophages have the ability to influence each phase of the angiogenic process, including modifying the local extracellular matrix, induction of endothelial cells to migrate or proliferate, and inhibition of vascular growth with formation of differentiated capillaries 147, thus facilitating the proliferation of endometrial cells and subsequently progressing to severe endometriosis. And neutrophils have been shown to release VEGF under inflammatory milieu and promote angiogenesis and the maturation of endometrial blood vessels in the early stage of endometriosis 126. Taken together, it is probable that IL-17 regulates ectopic lesions to create new blood vessels through both angiogenesis and vasculogenesis, so cutting off IL-17 might have a significant effect on blood supply of endometriotic lesions (**Figure 1**).

## Conclusions and perspectives

In conclusion, under the regulation of estrogen and ectopic microenvironment, such as hypoxia and cytokines, the secretion of IL-17 increases, which may be derived from endometrial stromal cells or from the production of Th17 differentiation. Then IL-17 further leads to the proliferation, growth and invasion of ectopic foci, promotes the immune escape of ectopic foci and the progression of endometriosis by recruiting and inducing M2 macrophage differentiation. In addition, IL-17 may well be involved in endometriosis-related infertility by motivating the secretion of activated macrophages, accentuating inflammation, influencing the stability of fetal-maternal interface and poisoning all stages of pregnancy. So as for the treatment, IL-17 may be placed as a candidate target molecule for novel treatment strategies of endometriosis, especially when endometriosis coexists with infertility. A moderate reduction in estrogen levels, inhibition of IL-17 inducer such as IL-23, IL-27, miR-342-3p and HIF-1, promotion of negative factors such as TGF-β1 and LncRNA H19, all are worth trying to block the source of IL-17 to intervene in this disease. Similarly, intercepting the downstream pathway of IL-17 may also affect the progression of endometriosis through decreasing IL-8 and Gro-α concentrations, normalizing the number of macrophages and neutrophils, and so on. Moreover, it is reported that the concentration of IL-17 is related to the degree of disease, which may be of certain value in evaluating therapeutic effect by the way of continuous monitoring of IL-17 level. However, the value of intervening IL-17A in the treatment of endometriosis is still to be studied.

## Figures and Tables

**Table 1:** Recent publications about IL-17, IL-17R or Th17 cells in patients with endometriosis and their correlation

Parameter	Distribution	Correlation	Reference
IL-17	PF↑	Negative correlation	60
IL-17	PF↑	No mention	63, 73, 74
IL-25	PF↑	No correlation	64
IL-17A	Plasma↑, PF↑	No mention	43, 61
IL-17	Serum↑	No mention	76
IL-17A	Serum↑, FF↑	No mention	77
IL-17A	Plasma↑, PF no difference	No mention	78
IL-17A-positive cells	Stroma and surrounding the vasculature in matched eutopic endometrium and ectopic lesion samples↑	No mention	78
IL-17	No difference	No correlation	84
IL-17	No difference	No correlation	85
IL-17/IL-10 ratio	PF↑, serum↑	No mention	85
IL-17/IL-23 ratio	PF↑, serum↑, periphery↑	No mention	85
IL-17F	EMMCs↑	No mention	79
IL-17RA	ESCs↑	No mention	62
IL-17RC	ESCs↑	No mention	79
IL-17A-positive cells	Endometriotic tissues↑	No mention	62
TH17 cells	PFMCs↑	No mention	62
TH17 cells	PF↑	Positive correlation	80, 81
IL-17 + TH17 cells	PFMCs↑	No mention	82
CD8^+^ T cells, CD56^+^ NK cells, IL-17 signalling pathway	Eutopic endometria↑	No mention	83

Note: Abbreviations: IL-17, interleukin 17; IL-17R, interleukin 17 receptor; Th17 cells, T helper 17 cells; PF, peritoneal fluid; FF, follicular fluid; EMMCs, mononuclear cells from endometriotic lesions; ESCs, endometriotic stromal cells; PFMCs, peritoneal fluid mononuclear cells.

## Abbreviations

IL-17: Interleukin-17
SLE: systemic lupus erythematosus
RA: rheumatoid arthritis
PF: peritoneal fluid
Th17: T helper 17
TGF-β: transforming growth factor-β
RORγt: retineic-acid-receptor-related orphan receptor gamma
NK: natural killer
FF: follicular fluid
ESCs: endometriotic stromal cells
PFMCs: peritoneal fluid mononuclear cells
EMMCs: mononuclear cells from endometriotic lesions
PB: peripheral blood
E2: 17β-estradiol
P4: progesterone
Erα: estrogen receptor alpha
TNF-α: tumor necrosis factor-α
RANTES: Regulated on Activation, Normal T cell Expressed and Secreted
MCP-1: Monocyte Chemotactic Protein-1
HRF: Histamine Releasing Factor
STAT3: signal transducer and activator of transcription 3
RORC: RAR-related orphan receptor C
c-Maf: c-musculoaponeurotic fibrosarcoma
Blimp-1: B lymphocyte-induced maturation protein-1
anti-BMP1: anti-bone morphogenetic protein 1
IER3: immediate early response gene
HIF-1: Hypoxia-inducible transcription factor-1
O_2_: oxygen
RASF-mediated: rheumatoid arthritis synovial fibroblasts-mediated
IFN-γ: interferon-γ
COX2: Cyclooxygensase-2
VEGF: vascular endothelial growth factor
G-CSF: granulocyte colony-stimulating factor
GM-CSF: granulocyte macrophage colony-stimulating factor
Gro-α: growth-related oncogene-α
EoSCs: endometrioma stromal cells
ROS: reactive oxygen species
SPM: small peritoneal macrophage
ECM: extracellular matrix
NOS2: nitric oxide synthase 2
NO: nitric oxide
EPCs: endothelial progenitor cells
